# Mucosal Immunity and the FOXO1 Transcription Factors

**DOI:** 10.3389/fimmu.2019.02530

**Published:** 2019-11-29

**Authors:** Dana T. Graves, Tatyana N. Milovanova

**Affiliations:** Department of Periodontics, School of Dental Medicine, University of Pennsylvania, Philadelphia, PA, United States

**Keywords:** bacteria, bone loss, forkhead, gingiva, immune, mucosa, periodontal disease, periodontitis

## Abstract

FOXO1 transcription factors affect a number of cell types that are important in the host response. Cell types whose functions are modulated by FOXO1 include keratinocytes in the skin and mucosal dermis, neutrophils and macrophages, dendritic cells, Tregs and B-cells. FOXO1 is activated by bacterial or cytokine stimulation. Its translocation to the nucleus and binding to promoter regions of genes that have *FOXO* response elements is stimulated by the MAP kinase pathway and inhibited by the PI3 kinase/AKT pathway. Downstream gene targets of FOXO1 include pro-inflammatory signaling molecules (TLR2, TLR4, IL-1β, and TNF-α), wound healing factors (TGF-β, VEGF, and CTGF) adhesion molecules (integrins-β1, -β3, -β6, α_v_β_3_, CD11b, CD18, and ICAM-1), chemokine receptors (CCR7 and CXCR2), B cell regulators (APRIL and BLYS), T-regulatory modulators (Foxp3 and CTLA-4), antioxidants (GPX-2 and cytoglobin), and DNA repair enzymes (GADD45α). Each of the above cell types are found in oral mucosa and modulated by bacteria or an inflammatory microenvironment. FOXO1 contributes to the regulation of these cells, which collectively maintain and repair the epithelial barrier, formation and activation of Tregs that are needed to resolve inflammation, mobilization, infiltration, and activation of anti-bacterial defenses in neutrophils, and the homing of dendritic cells to lymph nodes to induce T-cell and B-cell responses. The goal of the manuscript is to review how the transcription factor, FOXO1, contributes to the activation and regulation of key leukocytes needed to maintain homeostasis and respond to bacterial challenge in oral mucosal tissues. Examples are given with an emphasis on lineage specific deletion of *Foxo1* to explore the impact of FOXO1 on cell behavior, inflammation and susceptibility to infection.

Forkhead box-O (FOXO) transcription factors were first identified in Drosophila melanogaster (1). There are four members of this family in mammals, three of which (FOXO1, FOXO3, and FOXO4) have conserved sequence homologies while FOXO6 is more distantly related (2). FOXO proteins regulate cell survival and apoptosis, proliferation, energy metabolism, oxidative stress responses, and its mutations are closely linked to cancer formation (1, 3). FOXO1, FOXO3, and FOXO4 often have common target genes and function. However, there are differences that are related to interaction with different co-activators and co-repressors. For example, global *Foxo1* deletion in mice is embryonically lethal in contrast to global ablation of *Foxo3* or *Foxo4*, which is not. The biological functions of FOXOs can overlap but are not necessarily redundant. FOXOs act primarily as transcription factors following translocation to the nucleus but can sometimes have “off target” effects as co-regulators in the nucleus or by binding to other proteins in the cytoplasm (4).

FOXOs are controlled at several levels including expression, nuclear translocation, DNA binding and interaction with other proteins. FOXOs have four primary domains with the following functions: (a) DNA binding, (b) nuclear localization, (c) nuclear export, and (d) transactivation. FOXOs recognize two consensus response elements: a Daf-16 binding site (5′-GTAAA (T/C)AA) and an insulin-response element (5′-(C/A)(A/C)AAA(C/T)AA) (1). The core DNA sequence 5′-(A/C)AA(C/T)A is recognized by all FOXO-family members. FOXO post-translational modification involves acetylation, phosphorylation, ubiquitination, methylation, and glycosylation (1). The modifications affect nuclear translocation or exit from the nucleus, DNA binding, and interaction with co-repressors and co-activators (5). Kinases/phosphatases and acetylases/deacetylases modulate shuttling of FOXOs to and from the nucleus. FOXO1 nuclear localization and resulting transcriptional activity is downregulated by phosphorylation from insulin stimulation via the phosphoinositide-3-kinase/AKT pathway or conversely, up-regulated by phosphorylation at different aminoacids through RAS/mitogen-activated protein kinase activity (1). Deacetylation of FOXO1 typically enhances nuclear localization and activity while they are reduced by acetylation (6). Generally, the level of FOXO1 nuclear localization is proportional to its activity. However, we have found that high glucose increases FOXO1 nuclear localization but reduces induction of specific genes (TGF-β and VEGF) by reducing its binding to the promoter regions despite increased nuclear localization (7–10). In fact, FOXO1 can bind to specific molecules to act as part of a co-repressor or co-activator complex (11). In this regard it is not always simple to predict the impact of FOXO1 on a given activity since its function his highly modified by post-translational modification and interaction with other partners.

FOXOs play a key role in maintaining homeostasis and in adapting to environmental changes (2). Since FOXO1 is the best studied of the FOXO family it is the focus of this review. FOXO1 may have an important role in regulating several aspects of mucosal immunity by affecting dendritic cells (12), macrophage and neutrophil recruitment and activation (13–15), as well as T-helper cell and B-lymphocyte development and function (16–18). FoxO1 also affects immune responses by controlling cytokine production (19) and protecting hematopoietic stems cells from oxidative stress (20). In addition, FOXO1 regulates important aspects of keratinocyte function and potentially has a role in maintaining or repairing epithelial barrier function (21, 22). Surprisingly FOXO1 can have a specific effect under normal conditions and opposite effect in other conditions such those in diabetes and can have cell specific responses (21, 23). Thus, it is often difficult to predict the impact of FOXO1 and its role under various conditions. These studies demonstrate the complex nature of FOXO1 and its responsiveness to the cellular microenvironment, suggesting that it is highly regulated by epigenetic factors such as high glucose or those where oxidative stress is high. This is likely to be a fruitful area of future research.

## Periodontal Disease Pathogenesis

Periodontal disease is an inflammatory disease that is initiated by bacteria that forms a biofilm on the tooth surface and includes gingivitis consisting of gingival inflammation but not bone loss and periodontitis that leads to a net loss of bone (24–26). Periodontitis is recognized as the most prevalent lytic bone disorder in humans and the most common cause of tooth loss in adults in developed countries. In addition, periodontal disease, particularly periodontitis is linked to other chronic diseases such as rheumatoid arthritis, cardiovascular disease, and insulin resistance associated with type 2 diabetes (26). When bacteria or their products encounter leukocytes the host response is activated. Although periodontal disease is considered a destructive process it should be kept in mind that it represents corollary damage resulting from an effective host response that limits spread of bacteria (27–29). This concept is supported by findings that a combined *TLR2/TLR4* deletion that impairs the host response reduces periodontal bone resorption but increases systemic dissemination of oral bacteria (27). Another line of evidence that supports this conclusion is the limited colonization of gingival tissues by bacteria, indicative of the effectiveness of the host response in clearing bacteria despite the continual presence of bacteria in the gingival sulcus (28). However, when the host response is sufficiently compromised bacteria can invade the gingival tissues effectively (28). Further support comes from studies which demonstrate that there is very little damage caused directly by periodontal pathogens *in vivo* and that most of the damage occurs indirectly from the host response (29, 30). Thus, under typical conditions the bacteria are not sufficiently robust compared to the host defense and are prevented from colonizing gingival connective tissues and directly causing damage (27–29). A key component of the transition from gingivitis to periodontitis is the movement of inflammation from a sub-epithelial compartment toward bone (31). The proximity of inflammatory mediators to osteocytes/osteoblasts and PDL cells leads to the induction of RANKL by these cells as well as inhibition of coupled bone formation and periodontal bone loss (32, 33). Several mechanisms may facilitate this transition including a bacterial dysbiosis, bacterial penetration to connective tissue, ineffective removal of bacteria or their products, inadequate function of several cell types including neutrophils and dendritic cells, lack of adequate stimulation of Th2 and T-regulatory lymphocyte responses, hyper-activation of a Th1 and Th17 responses and failure to down regulate inflammation through various mechanisms (34–41). The importance of an adequate host response to bacterial challenge has been shown by increased susceptibility to periodontitis in mice with genetic deletion of specific genes that regulate leukocyte recruitment such as *Icam-1, P-selectin, Beta2-integrin/CD18*; recognition of bacteria by *TLR2, TLR4, Lamp-2*; immune modulation by *Cxcr2, Ccr4, IL-10, OPG, IL1RA, TNF-*α *receptor, IL-17 receptor, Socs3, Foxo1*; and deletion of genes that encode proteolytic enzymes including *Mmp8 and Plasmin* (42). The adaptive immune response produces inflammatory mediators that stimulate apoptosis in osteoblasts through a mechanism involving activation of FOXO1 in osteoblasts and suppression of coupled bone formation, an important component of periodontal bone loss (19, 39).

## Keratinocytes and FOXO1

An epithelial barrier separates the gingival connective tissue from the external environment and protects it from bacterial colonization (43). It consists primarily of keratinocytes, which are separated from the connective tissue by a basement membrane. Epithelial cells produce cell to cell junctions, inflammatory cytokines, and elaborate anti-microbial peptides that limit bacterial invasion (44). *In vitro*, oral bacteria are able to pass the epithelial barrier via different paths: *Porphyromonas gingivalis* (*Pg*) can invade by intracellular spread from epithelial cell to epithelial cell, *Aggregatibacter* actinomycetemcomitans (*Aa*), and Fusobacterium nucleatum (*Fn*) move between epithelial cells. In contrast, Streptococcus gordonii (*Sg*) is predominantly associated with the superficial cell layer (45). Transcription factors such as FOXO1 play important roles the response of keratinocytes to perturbation by bacteria or wounding (22, 46–48). *Porphyromonas gingivalis* stimulates an increase in FoxO1 expression and has multiple effects on gingival epithelium including a loss of barrier function (47). FOXO1 is needed for keratinocytes to maintain expression of integrins beta-1, beta-3, and beta-6, which may be critical to maintaining barrier function (47). FOXO1 has also been shown to mediate keratinocyte responses to bacteria. For example, FOXO1 mediates *Porphyromonas gingivalis*-stimulated expression of antioxidants (catalase, superoxide dismutase, and peroxiredoxin 3) (48). *Porphyromonas gingivalis* activates FOXO1 by inducing the production of ROS, which in turn stimulates JNK activation and presumably stimulates FOXO1 nuclear localization (48). Surprisingly, knockdown of FOXO1 under basal conditions increases IL-1β production suggesting that FOXO1 in the absence of an inflammatory stimulus acts to restrain inflammation (48). Short-term exposure of keratinocytes to *Porphyromonas gingivalis* reduces apoptosis, while long-term exposure increases keratinocyte cell death. *Porphyromonas gingivalis*-stimulated apoptosis under the latter conditions is FOXO1 dependent (47).

Several classes of genes expressed by keratinocytes are FOXO1 dependent including keratin-1, -10, -14, and involucrin, which are expressed in differentiated keratinocytes (47). Similarly, genes that maintain barrier function such as integrin beta-1, -3, and -6 are FOXO1 regulated (47). Thus, FOXO1 affects several genes that affect keratinocyte behavior that potentially modulate barrier function and are needed for cell to cell adhesion or adhesion to matrix proteins.

**Figure 1:**
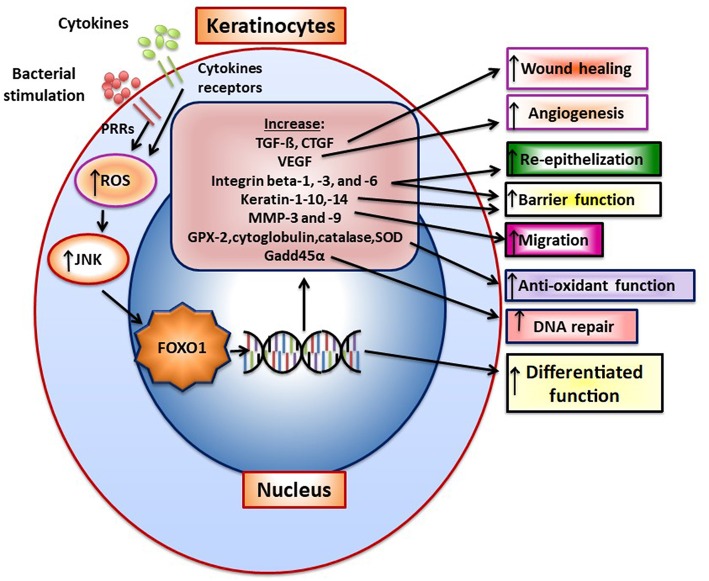
FOXO1 is activated in keratinocytes to induce gene expression that modulates keratinocyte behavior. FoxO1 induces the expression of genes that affect keratinocyte function such as TGF-ß, VEGF, CTGF, integrins beta-1, -3, -6, Keratin-1, -10, -14, antioxidants GPX-2, cytoglobin, catalase and superoxide dismutase, and a DNA repair enzyme, Gadd45α. Bacteria through pattern recognition receptors or cytokine stimulation induces formation of reactive oxygen species that activate components of the MAP kinase pathway such as JNK, which stimulate FoxO1 nuclear localization where FoxO1 modulates gene transcription.

Upon wounding keratinocytes respond quickly to re-epithelialize the wounded surface. Wound healing increases FoxO1 nuclear-localization in keratinocytes to promote re-epithelialization (49). TGF- β is quickly released upon wounding. FoxO1 mediates the effect of TGF-β on keratinocytes (50) and FoxO1 is needed to upregulate TGF-β expression in keratinocytes during wound healing suggesting a reciprocal relationship (49). TGF-β promotes epithelial migration to cover the wound surface and without adequate TGF-β signaling re-epithelialization is compromised. Other components in epithelial cell migration are induced by FoxO1 during wounding including integrin-β3 and -β6 and MMP-3 and -9. Furthermore, FOXO1 promotes re-epithelialization by increasing resistance to oxidative stress through the induction genes with anti-oxidant activity [e.g., glutathione peroxidase 2 (GPX-2) and cytoglobin] and which repair damaged DNA (e.g., growth arrest and DNA damage inducible 45α, GADD45α) (49). This is significant since high levels of ROS interfere with keratinocyte function and compromise re-epithelialization (49). In the absence of FOXO1 keratinocyte apoptosis is also increased during healing and associated with increased oxidative stress (49). Figure 1 presents a summary of FOXO1 downstream gene targets and their potential effect on keratinocyte function.

The above functions of FOXO1 have been shown to occur in re-epithelialization of the wounded skin. Studies have also demonstrated that FoxO1 is required for mucosal re-epithelialization. FOXO1 expression in keratinocytes is needed for repair of injured mucosa under normal conditions. In a diabetic environment FOXO1 has the opposite effect as shown by improved migration of mucosal keratinocytes and improved re-epithelialization in diabetic mice with keratinocyte specific *Foxo1* ablation (7). A potential mechanism involves the altered expression of FOXO1 downstream target genes based on glycemic levels. For example, hyperglycemia *in vivo* and in high glucose *in vitro* increase FOXO1 interactions response elements in chemokine CCL20 and interleukin-36γ promoters that increase transcription in a FOXO1-dependent manner. High levels of CCL20 and IL-36γ stimulated by high glucose interfere with keratinocyte migration. Thus, in high glucose FOXO1 fails to induce TGF-β, which can enhance keratinocyte migration and instead causes excessive production of CCl20 and IFNγ, which inhibit migration (7). Thus, the glucose environment changes the activity of FOXO1 so that it promotes mucosal epithelialization under normal conditions but causes a shift in its induction of downstream targets that at to inhibit re-epithelialization.

FoxO1 activity in keratinocytes may also affect the underlying connective tissue. It has been shown that expression of VEGF in keratinocytes is dependent on *Foxo1* (9) since its deletion reduces VEGF expression and keratinocyte-stimulated angiogenesis in the underlying connective tissue *in vivo*. Furthermore, FOXO1 induces TGF-β and CTGF in keratinocytes and *Foxo1* ablation in keratinocytes reduces the number of mesenchymal stem cells and fibroblasts *in vivo* (51). These results suggest that FOXO1 is an important transcription factor in epithelium that participates in connective healing by the production of growth factors such as VEGF, TGF-β, and CTGF. In contrast, the role of FOXO1 in organizing the mucosal keratinocyte response to microbial challenge is not as well understood although it is evident that FOXO1 is induced by bacterial challenge (44, 48). Future research may provide insight into the potential regulation of barrier function by FOXO1 as well as clarify its regulation of the inflammatory response of keratinocytes to microbes *in vivo* and potential anti-microbial functions.

## Dendritic Cells and FOXO1

Dendritic cells (DCs) are antigen-presenting cells, which capture, process and present antigens to lymphocytes to initiate and regulate the adaptive immune response (38). Microbial products can stimulate dendritic cells (DC) through toll-like receptors (TLRs) to enhance T-cell activation (52, 53). Trafficking of DC through lymphatic vessels is an essential aspect of protection by clearing bacteria and promoting protective immune responses (54). There is increased DC trafficking to lymph nodes and to the gingiva in response to the accumulation of dental plaque (55) and a decrease following periodontal treatment (56). In one of the few cause and effect studies, ablation of Langerhans cells and Langerhans^**+**^ dendritic cells resulted in reduced numbers of Tregs, elevated production of RANKL, and enhanced alveolar bone loss during experimental periodontitis (57). In contrast, a more specific deletion of mucosal Langerhans cells had no effect on *Porphyromonas gingivalis* induced periodontitis but did enhance the production of Th17 cells (58). The authors in the latter report suggest that neither Langerhans cells nor Th17 cells play a major role in *Porphyromonas gingivalis* induced periodontal bone loss in the mouse model.

**Figure 2:**
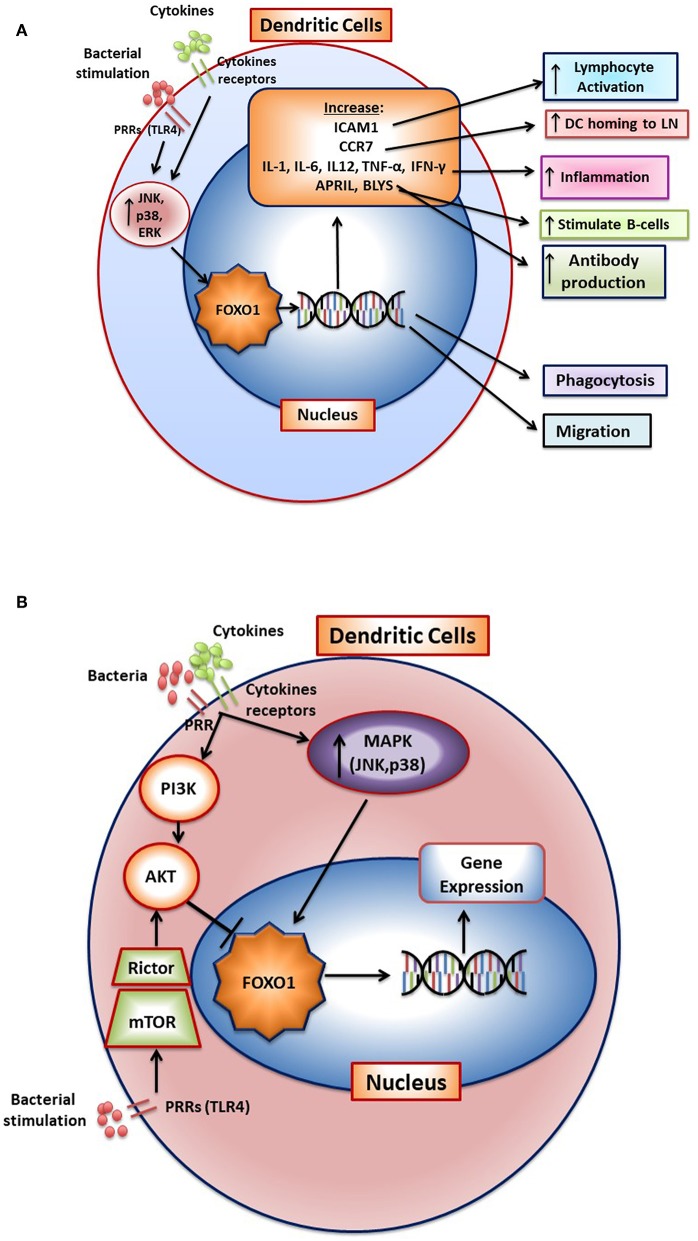
FOXO1 regulates activation and function of dendritic cells. **(A)** FoxO1 nuclear localization is stimulated by activation of the MAP kinase pathway (JNK, p38, and ERK). In the nucleus FOXO1 affects DC function by increasing expression of genes such as ICAM1, CCR7, APRIL, BLYS, IL-1-β, IL-6, IL12, IFN-γ, TNF-α, and αvβ3. The change in gene expression promotes DC homing to the lymph nodes, antigen presentation, DC activation of T-and B- cells, and inflammation. **(B)** Cytokine receptors and pattern recognition receptors such as toll-like receptors (TLRs) stimulate activation of FOXO1 through the MAP kinase pathway. Activated FOXO1 can bind to the promoter region of target genes and regulate transcription. AKT is a major downstream target of PI3K that functions as a negative regulator of FOXO1. Stimulation of mTOR activates AKT to inhibit FoxO1 activity, which has been proposed to prevent a hyperinflammatory response.

Bacteria activate FOXO1 in DC by inducing its nuclear localization through the MAPK pathway (12). FOXO1 affects several aspects of DC function. FOXO1 directly or indirectly participates in several aspects of DC stimulation of T- and B-lymphocytes through its effect on bacterial phagocytosis, lymphocyte migration, and homing as well as DC-lymphocyte binding. It is needed for DC phagocytosis of bacteria as *FOXO1* specific deletion in DCs inhibits bacterial phagocytosis (12). In addition, it affects DC migration and DC binding to lymphocytes (12). DC homing to lymph nodes is a key early aspect of the adaptive immune response. After engaging bacteria, DCs in the mucosa move to regional lymph nodes where they interact with lymphocytes. FOXO1 plays a key role in DC homing to lymph nodes through upregulation of CCR7. CCR7 is a chemokine receptor that responds to ligands expressed in lymph nodes to direct DC-lymph node homing. In addition, FOXO1 upregulates expression of ICAM-1. ICAM-1 is needed for DC binding to lymphocytes and formation of an immune synapse that activates lymphocytes. FOXO1 is able to bind to response elements in the promoters of ICAM-1 and CCR7 consistent with direct transcriptional regulation (12). Transfection with ICAM-1 and CCR7 expression vectors rescues impaired DC function *in vivo* and *in vitro* in *Foxo1* deleted DCs demonstrating their functional significance. FOXO1 downstream gene targets and their impact on DC function are summarized in Figure 2A. The linkage between FOXO1, DC and the capacity of DC to stimulate adaptive immunity is reinforced by evidence that bacteria-specific antibody production *in vivo* is impaired by lineage specific *Foxo1* ablation in DC (12). When FOXO1 is over-expressed *in vitro*, DCs produce high levels of IL-12, IL-6, and TNF-α and when *Foxo1* is deleted DCs have reduced capacity to produce inflammatory cytokines (59). Thus, FOXO1 regulates expression of inflammatory cytokines in DCs as does AKT1, which inhibits FOXO1 activity (58–64).

Bacteria and their products increase FOXO1 expression and activation in DCs by signaling through TLRs (59–61). Oral infection stimulates DC migration to cervical lymph nodes and induces antibody production *in vivo* (59). Recent experiments have demonstrated that periodontitis lesions are characterized by neutrophils and T-helper cells (36, 65). Deletion of *Foxo1* specifically in DCs reduces DC homing to lymph nodes induced by periodontal pathogens and reduces the production of specific antibody in response to their oral inoculation (59). The up-regulation of adaptive immunity is in part dependent upon FOXO1 regulation of APRIL and BLYS (see Figure 2A). This is significant since APRIL and BLYS are needed for stimulation of B-cells to form plasma cells (59). In addition, oral infection stimulates DC migration to mucosal epithelium. This migration is reduced by DC-specific *Foxo1* ablation (59). Interestingly, reduced DC homing to lymph nodes and periodontal tissues caused by lineage specific *Foxo1* deletion in DCs increases periodontal inflammation and susceptibility to periodontitis (59). The enhanced susceptibility is most likely through reduced humoral immunity, suggesting an important protective role for adaptive immunity in protecting periodontal tissues from microbial dysbiosis. The decreased antibody protection against bacteria in turn, may lead to an increased innate immune response that mediates periodontal tissue destruction. The latter is supported by findings that specific ablation of *Foxo1* in DC is linked to increased IL-1β and IL-17 levels, greater RANKL expression and osteoclast formation and more bone loss (59). In addition, DC-specific ablation of *Foxo1* in aged mice reduces anti-*Porphyromonas gingivalis* IgG1 and is associated with greater periodontal bone loss (62).

Stimulation of DC with bacteria such as *Porphyromonas gingivalis* or LPS induces a significant increase in FOXO1 activation as reflected by increased nuclear localization. Moreover, bacteria-induced FOXO1 nuclear localization is blocked by inhibitors of p38, JNK, and ERK. The inhibition of all three MAP kinase components is more effective than any one of them alone. However, the regulation of FOXO1 activity is more complex. In addition to stimulating FOXO1, TLR4 signaling can indirectly inhibit FoxO1 activation (63). In this scenario, LPS stimulation in DC activates mTOR and subsequently stimulates AKT, which inhibits FOXO1 activity. The activation of AKT in dendritic cells may prevent a hyperinflammatory response by deactivating FOXO1 (60). In addition, AKT has been reported to induce dendritic cell proliferation and survival (64). The activation of FOXO1 through the MAP kinase pathway and inhibition of FOXO1 through mTOR induced AKT signaling is shown in Figure 2B. These studies suggest that FOXO1 and AKT1 interact to modulate inflammatory responses in DC *in vivo*. It remains to be proven whether this occurs *in vivo* and whether AKT1 functions in DC to down-regulate FOXO1 or whether it primarily modulates DC function through phosphorylation of other downstream targets such as mTor or GSK-3.

## Lymphocytes and FOXO1

Lymphocytes play important protective and destructive roles in periodontitis (25). B-lymphocytes are the predominant leukocyte in chronic inflammatory periodontal lesions and differentiate to plasma cells that produce antibody (36). There is no consensus on whether the development of antibodies in periodontitis is protective, although some studies have shown that a deficient Th2 response is associated with increased susceptibility to periodontitis (25). Similarly, the deletion of B-cells in mice have led to inconsistent results (36). Increased Th2 and Treg lymphocyte production is generally associated with resistance to periodontitis or resolution of periodontal inflammation (66). Th2 lymphocytes produce anti-inflammatory cytokines such as IL-4 and IL-10 and antibodies that may be protective (25). T-regulatory lymphocytes suppress inflammation by production of cytokines such TGF-β and IL-10. In experimental periodontitis adoptive transfer of Tregs inhibits periodontal disease susceptibility (67). Development of chronic periodontitis is linked to Th1 lymphocytes that produce IFNγ and IL-1-β and Th17 lymphocytes that produce IL-17A (68). Inflammation is problematic because it inhibits coupled bone formation that occurs after an episode of periodontal bone loss (39). In addition, RANKL, which plays an essential role in periodontal bone resorption has other properties besides stimulating osteoclastogenesis and is needed to form germinal centers in lymph nodes and may be important in enhancing formation of Tregs in inflamed bone (69).

**Figure 3:**
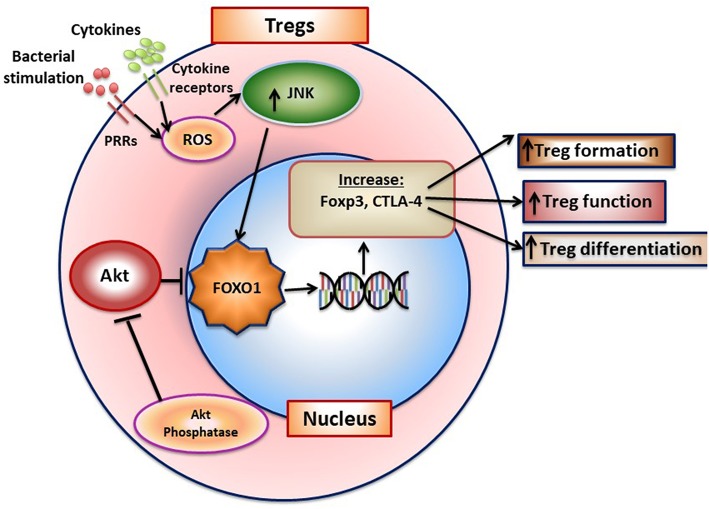
FOXO1 regulates formation of T-regulatory cells. FoxO1 induces Foxp3, CTLA-4, IL-10, and TGF-β to enhance Treg formation and function. Activation of AKT phosphatase blocks AKT activation, which functions to increase FOXO1 nuclear localization to maintain Treg function.

FOXO1 plays an important role in adaptive immunity. FOXO1 affects lymphocyte development, homing, cytokine expression, and gene recombination (70). It modulates the formation of Tregs B-lymphocytes and is instrumental in maintaining hematopoietic stem cells (71, 72). Antibody class-switch by B-cells is also FOXO1 dependent (73). It exerts these effects through transcriptionally inducing key downstream target genes including L-selectin and sphingosine-1-phosphate receptor 1 (S1pr1) for homing and IL-7 receptor-α that promotes survival of naive T-cells. FOXO1 promotes the formation of T-regulatory cells and B-lymphocytes (71, 72). The formation and function of Tregs is negatively affected by *Foxo1* ablation as mice with lineage specific *Foxo1* deletion have substantially reduced Tregs and those that are formed have diminished viability and function (70, 74). Furthermore, in the absence of FOXO1 TGF-β stimulated Tregs formation is reduced and Th1 cells increased (75). FOXO1 binds to the *Foxp3* and cytotoxic T-lymphocyte antigen 4 (*Ctla4*) promoters to induce their transcription (70). AKT inhibits FOXO1 and reduced AKT activity is needed for FOXO1 to induce Treg formation. Moreover, inflammatory conditions may stimulate the PI3K/AKT pathway and inhibit formation of Tregs (76). A summary of FOXO1 and its role in Tregs is shown in Figure 3. Studies of FOXO1 function in dendritic cells demonstrate that FOXO1 protects against bacteria induced periodontitis through upregulation of dendritic cell activity. However, they have not yet addressed whether FOXO1 induces formation and activity of specific CD4^+^ T-helper cell phenotypes or the formation and activity of CD8^+^ lymphocytes that may affect resistance or susceptibility to periodontal disease.

## Monocytes/Macrophages and FOXO1

**Figure 4:**
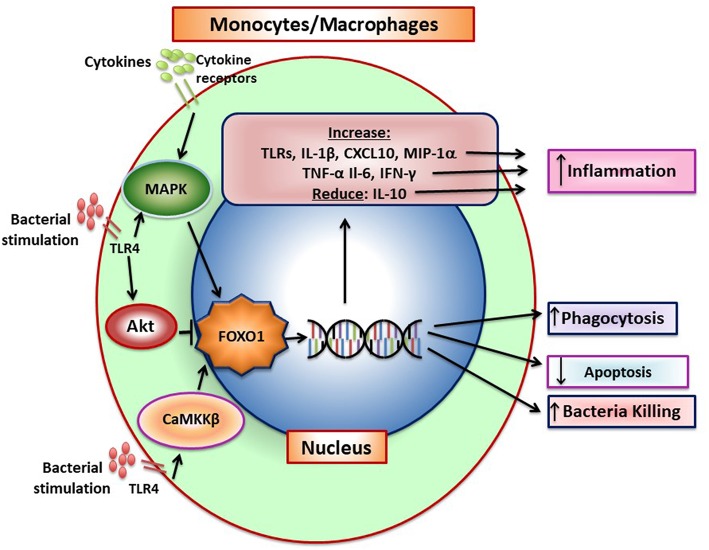
FOXO1 increases production of inflammatory mediators by monocytes/macrophages. Pattern recognition receptors and cytokine receptor stimulation induces FOXO1 activity. FoxO1 increases expression of TLRs, IL-1β, CXCL10, MIP-1α, TNF-α, IL-6, and IFN-γ and can also reduce IL-10, which combined, increase inflammation. Pattern recognition receptors such as TLR4 can induce CaMKKβ that leads to increased FOXO1 nuclear localization and activation, which has been linked to increased bacterial killing. In addition, FOXO1 can act to increase phagocytosis and reduce apoptosis. There is conflicting data on FOXO1 and macrophage polarization. In tumor-associated macrophages FOXO1 is reported to increase the M1 phenotype but in asthma to increase the M2 phenotype.

Monocytes are mobilized to the peripheral tissue by infection. Macrophages exist in different forms, classically pro-inflammatory M1 macrophages and anti-inflammatory/pro-healing M2 macrophages (77). M1 macrophages are generated when exposed to IFNγ, TNF–α, IL-1, and IL-6 while M2 macrophages are induced by IL-4, IL-10, and IL-13 (77). It is thought that there is a continuum between M1 and M2 macrophages, that most are not purely M1 or M2 and that they can be programmed to change M1 and M2 phenotypes. A third M3 macrophage phenotype has been proposed that results from incomplete macrophage reprogramming (77). These intermediate M3 polarization states may prevent an excessive response resulting from macrophage polarization. FOXO1 and its function in macrophages is summarized in Figure 4.

When exposed to bacteria, M1 macrophages secrete inflammatory cytokines that directly or indirectly stimulate osteoclast formation as well as proteases that degrade connective tissue matrix. IL-1β and TNF-α produced by monocytes/macrophages are highly expressed during induction of periodontal disease (78). The conversion from the gingivitis to periodontitis and recruitment of inflammatory cells in close proximity to bone is blocked by IL-1 and TNF-α blockers demonstrating the importance of these macrophage products in the destructive process (31). However, at later stages macrophage also contribute to the resolution of inflammation through the removal of apoptotic neutrophils and stimulate repair (37). This reflects the conversion from the M1 to the M2 phenotype. FOXO1 and its function in macrophages is summarized in Figure 4.

TLRs stimulate FOXO1 activity in macrophages (79). FoxO1 activation in macrophages is pro-inflammatory as it increases IL-1β production (80). FOXO1 binds to response elements in the IL-1β promoter to increase transcriptional activity (80). FOXO1 also binds to DNA response elements of a number of genes in the TLR signaling pathway including TLR4 itself to enhance inflammation (14). Thus, FOXO1 can enhance TLR4 stimulated expression of IL-1β, Cxcl10, and MIP1α through upregulation of molecules in the TLR4 pathway as well as induce their transcription directly. FOXO1 may also protect macrophages from apoptosis to increase their survival in an inflammatory environment and enhance inflammation (81). Hyperglycemia may further increase the macrophage inflammatory phenotype by reducing the capacity of FoxO1 to stimulate IL-10 expression (13).

The effect of FOXO1 on macrophage polarization has been controversial and may depend upon specific conditions. *Foxo1* deletion in myeloid cells has been shown to reduce M1 and increases M2 polarization in macrophages (79). As a result, these experiments suggest that FOXO1 in macrophages promotes M1 polarization in concert with its pro-inflammatory function. Consistent with this hypothesis, tumor associated macrophages that have reduced FOXO1 expression exhibit increased M2 polarization, which is thought to enhance tumor growth (82). However, it has also been reported that in response to LPS, M2 macrophages exhibit increased FOXO1 expression and FOXO1 binds to the IL-10 promoter in M2 macrophages more efficiently than it does in M1 macrophages (13). Thus, under these experimental conditions FOXO1 promotes M2 macrophage formation by inducing IL-10, Arg1, Fizz1, and interleukin-13 receptor alpha 1 (IL-13Ra1) (13).

FOXO1 activity may affect macrophage function by modulating autophagy (83). Autophagy has been proposed as a mechanism by which macrophages deal with intracellular pathogens to enhance their killing (84). When macrophages are challenged with *E. coli* there is an increase in cellular calcium levels that promotes autophagy and anti-bactericidal activity that is stimulated by calcium/calmodulin dependent protein kinase β (CaMKKβ). CaMKKβ leads to increased FOXO1 nuclear localization (83). When *Foxo1* is knocked down there is a significant reduction in autophagy.

Although FOXO1 is pro-inflammatory in macrophages it may also limit a hyper-inflammatory response by inducing AKT that in turn inactivates FOXO1 (14). It has been proposed that TLR4 activation stimulates PI3 kinase activity, which in turn phosphorylates AKT to induce FOXO1 phosphorylation and its transit from the nucleus to the cytoplasm to deactivate it. A similar mechanism has been proposed in dendritic cells as described above and in Brown et al. (60). Taken together, it is possible that insulin has an anti-inflammatory effect by inducing AKT activity and inhibiting FOXO1. FOXO1 may also augment the innate immune response by affecting myeloid cells. Genetic ablation of *Foxo1, Foxo3*, and *Foxo4* in myeloid cells results in an expansion of granulocyte/monocyte progenitors (81). The increased formation of these progenitors is likely due to an inhibitory effect of FOXOs on cell-cycle progression that is lost when the FOXOs are deleted. The precise role of FOXO1 in macrophages has not been settled and it may depend on the conditions tested. The interpretation of these studies is also limited by a lack of *in vivo* studies with more specific deletion of *Foxo1* in monocytes/macrophages. The role of FOXO1 in macrophage function is summarized in Figure 4.

## Neutrophils and FOXO1

Neutrophils are the predominant leukocyte recruited to the gingiva by bacteria or their products that have crossed the epithelial barrier and entered the connective tissue. One hypothesis for the development of periodontal disease is an inadequate neutrophil defense that that leads to greater inflammation and periodontal destruction (85). Neutrophil polymorphonuclear leukocytes (PMNs) phagocytose and kill microbes and remove subcellular particles (85). Following an acute inflammatory response the removal of apoptotic neutrophils is needed to resolve inflammation; a failure to remove apoptotic neutrophils interferes with resolution and leads to prolonged inflammation (86). Neutrophils and their products are responsible for much of the destruction of periodontal connective tissue and may also contribute to loss of epithelial barrier by inducing micro-ulceration.

**Figure 5:**
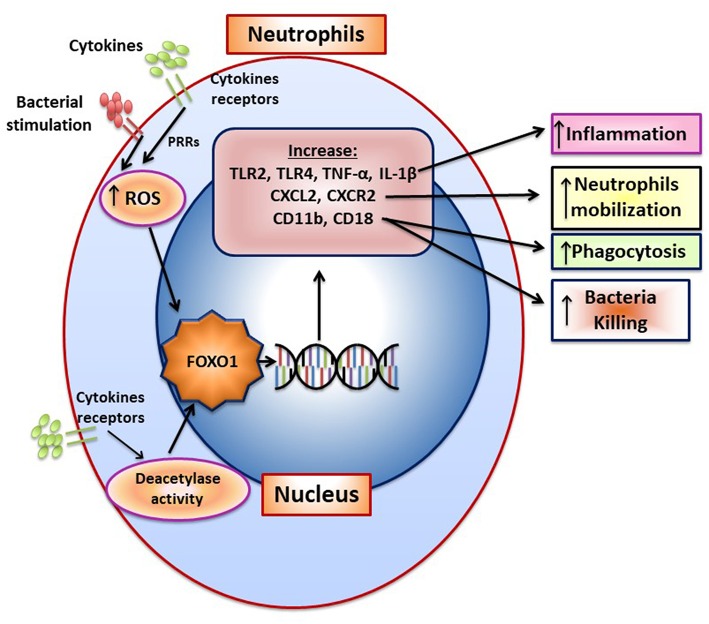
FOXO1 increases neutrophil mobilization and function. The formation of ROS and NOS induced by pattern recognition and cytokine receptors increase FOXO1 nuclear localization and activity. FOXO1 nuclear localization is also stimulated by deacetylation due to deacetylase activity. FOXO1 increases expression of TLR2, TLR4, TNF, and IL-1β to increase inflammation. CXCL2 and CXCR2 are induced by FOXO1 and are associated with enhanced neutrophil mobilization resulting from infection. CD11b and CD18 are integrins that are induced by FOXO1 and facilitate migration, phagocytosis, and bacterial killing.

Bacteria induce FOXO1 activation in neutrophils through TLR2 and TLR4, which is linked to FOXO1 deacetylation and stimulation by reactive oxygen species (15). TLR2 and TLR4 signaling stimulate FOXO1 nuclear localization. The nuclear localization is dependent on formation of ROS since inhibitors that block formation of ROS or NOS reduce FOXO1 activation. Moreover, bacteria-induced FOXO1 nuclear localization is also dependent on deacetylation since Sirt1 and histone deacetylase inhibitors reduce FOXO1 nuclear localization. The latter is consistent with findings that acetylation and phosphorylation of FOXO1 at specific sites block its translocation to the nucleus (6). In addition to responding to TLR2/TLR4 signaling, FOXO1 may act as a feed forward loop to enhance inflammation. This is based on findings that over-expression of FOXO1 increases upregulation of TLR2/4 and enhances neutrophil mediated inflammation by increasing inflammatory cytokine expression (e.g., TNF and IL-1) (15). An important component in the response of neutrophils to infection is the mobilization of neutrophils from bone marrow to the vasculature and migration to infected sites (87). *Foxo1* ablation in neutrophils interferes with neutrophil mobilization, which is mechanistically linked to FOXO1 induction of the chemokine CXCR2 (15). When *Foxo1* is ablated in neutrophils there is a significant reduction in neutrophil mobilization that coincides with reduced bacterial clearance and a reduced capacity of neutrophils to phagocytose and kill bacteria *in vitro* (15). The impact of FOXO1 on phagocytosis is tied to its regulation of CD11b, which along with CD18 captures bacteria to facilitate phagocytosis (15). A summary of FOXO1 downstream target genes and their impact on neutrophil function is shown in Figure 5.

In summary, FOXO1 is activated by bacteria or their products in several sub-classes of myeloid cells and lymphocytes as well as keratinocytes. These cell types are important in mucosal immunity. FOXO1 has the potential to play an important role in maintaining homeostasis in periodontal tissues and in the response to bacterial challenge. Alterations in FOXO1 function have a significant effect of periodontal disease susceptibility and due to FOXO1 regulation of leukocyte function. These studies indicate that FOXO1 plays an important role in the host defense and suggest potential mechanisms through up regulation of cellular activity. A limitation of many of the above studies is lack of lineage-specific demonstration of *Foxo1* deletion in each cell type or sub-set to better define its activities.

## Author Contributions

DG conceived this review. DG wrote the first draft of the manuscript and DG and TM edited it. Both authors contributed to manuscript revision, read, and approved the submitted version.

### Conflict of Interest

The authors declare that the research was conducted in the absence of any commercial or financial relationships that could be construed as a potential conflict of interest.

## Abbreviations

FOXO 1: Forkhead box protein O1
TNF alpha: Tumor Necrosis factor alpha
TGF beta: transforming Growth factor
VEGF: Vascular Endothelial Growth factor
CTGF: Connective Tissue Growth Factor
APRIL: A-proliferation-inducing ligand
BLYS: B-lymphocyte Stimulator
ICAM-1: Intercellular Adhesion Molecule
GPX2: Glutathione peroxidase 2
GADD45a: Growth Arrest And DNA Damage Inducible Alpha
CCR7-C: C chemokine receptor type 7
Il-6: Interleukin 6
RANKL: Receptor activator of nuclear factor kappa-B ligand
DCs: Dendritic cells
Ly: Lymphocyte antigen
ROS: Reactive oxygen species
HO-1: Heme oxygenase 1
PD-L1: Programmed death-ligand 1
TLR: Toll like receptor
PDL-cells: periodontal ligament cells.
